# On the influence of structural and chemical properties on the elastic modulus of woven bone under healing

**DOI:** 10.3389/fbioe.2024.1476473

**Published:** 2024-10-01

**Authors:** Pablo Blázquez-Carmona, Juan Mora-Macías, Antonia Pajares, Álvaro Mármol, Esther Reina-Romo

**Affiliations:** ^1^ Escuela Técnica Superior de Ingeniería, Universidad de Sevilla, Sevilla, Spain; ^2^ Escuela Técnica Superior de Ingeniería, Universidad de Huelva, Huelva, Spain; ^3^ Departamento de Ingeniería Mecánica, Energética y de los Materiales, Universidad de Extremadura, Badajoz, Spain

**Keywords:** woven bone, nanoindentation, micro-CT, Raman sprectroscopy, composition, distraction osteogenesis, elastic modulus, power law

## Abstract

**Introduction:**

Woven bone, a heterogeneous and temporary tissue in bone regeneration, is remodeled by osteoblastic and osteoclastic activity and shaped by mechanical stress to restore healthy tissue properties. Characterizing this tissue at different length scales is crucial for developing micromechanical models that optimize mechanical parameters, thereby controlling regeneration and preventing non-unions.

**Methods:**

This study examines the temporal evolution of the mechanical properties of bone distraction callus using nanoindentation, ash analysis, micro-CT for trabecular microarchitecture, and Raman spectroscopy for mineral quality. It also establishes single- and two-parameter power laws based on experimental data to predict tissue-level and bulk mechanical properties.

**Results:**

At the macro-scale, the tissue exhibited a considerable increase in bone fraction, controlled by the widening of trabeculae. The Raman mineral-to-matrix ratios increased to cortical levels during regeneration, but the local elastic modulus remained lower. During healing, the tissue underwent changes in ash fraction and in the percentages of Calcium and Phosphorus. Six statistically significant power laws were identified based on the ash fraction, bone fraction, and chemical and Raman parameters.

**Discussion:**

The microarchitecture of woven bone plays a more significant role than its chemical composition in determining the apparent elastic modulus of the tissue. Raman parameters were demonstrated to provide more significant power laws correlations with the micro-scale elastic modulus than mineral content from ash analysis.

## 1 Introduction

During skeletal tissue differentiation processes such as distraction osteogenesis, fracture healing, adaptation to skeletal implants, bone development and regeneration, the structure and composition of the mineralized bone tissue, referred to as woven bone or immature bone, continually change due to growth and responses to the tissue environment, including physical, chemical, and mechanical factors (Wehrle et al., 2014; Tourolle Né Betts et al., 2020; Mora-Macías et al., 2021; Blázquez-Carmona et al., 2021a; Paul et al., 2022). The organic and inorganic matrices of woven bone are highly dynamic and undergo alterations in their structure and composition (Isaksson et al., 2010; Turunen et al., 2011; Martínez-Reina et al., 2018; Reznikov et al., 2018). The inorganic phase is primarily composed of nonstoichiometric hydroxyapatite crystals. This mineral content is deposited in two phases: an initial phase in which 70% of the final mineral content is incorporated within a few weeks, followed by a second phase in which the mineral content increases over months and years (Roschger et al., 2020). The organic phase is predominantly composed of type I collagen, along with various biomacromolecules, including proteoglycans and non-collagenous proteins (Boskey and Robey, 2013). During the initial stages of healing, naïve collagen fibers are randomly oriented due to their rapid formation. As the maturation progresses, these fibers increase in density, reorient, and structurally mature (Blázquez-Carmona et al., 2022). This maturation is understood as the process in which the fibers are crosslinked and packaged to accommodate mineralization. These microstructure arrangements result in a time-dependent mechanical behavior of the tissue, with initial stiffness much lower than that of the matured cortical and trabecular bones.

The microstructure and composition of bone play crucial roles in its macroscopic structural and mechanical behavior. Their relationship has been extensively studied in cortical and trabecular bones (Schaffler and Burr, 1988; Rho et al., 1995; Sevostianov and Kachanov, 2000; Budyn et al., 2012). It has been shown that the elastic modulus of the mature bones strongly depends on mineral content, while toughness correlates with the quality of the collagen matrix (Zioupos and Currey, 1998). Correlations between the elastic modulus and the bone mineral density have been established to estimate mechanical properties from the image-based measurements of the bone mineral density at both the organ and tissue scales (Nobakhti and Shefelbine, 2018). At the organ level, the elastic modulus (*E*) is related to the apparent mineral density ρ , mineralized bone mass/bulk volume, through a power law relationship $\left(E\;\propto \;\rho^{b}\right)$ . For cortical bones, b ranges between 4 and 7.4 (Schaffler and Burr, 1988; Currey, 1988) and for trabecular bones, it is normally lower, b = 1.27-2.57 (Rice et al., 1988; Keller, 1994; Bouxsein and Radloff, 1997), indicating lower apparent stiffness. One limitation of these models is that they do not distinguish the influence of bone volume fraction from ash fraction. Therefore, at the macro- level, these correlations are improved when porosity (Schaffler and Burr, 1988; Currey, 1988) or fabric orientation are included (Hodgskinson and Currey, 1993; Martin and Boardman, 1993; Nicholson et al., 1997). However, this relationship is weak at the tissue level, likely due to the effect of microstructural features at small length scales (Nobakhti and Shefelbine, 2018). In cortical bone, hierarchical micromechanical models have been also developed to explain how mechanical interactions between components at different observation scales regulate the tissue’s effective elastoplasticity (Fritsch and Hellmich, 2007; Hamed et al., 2010; Kumbolder et al., 2024). However, they traditionally assume invariant structural and mechanical properties of these elementary components, including the collagen and hydroxyapatite (Hellmich et al., 2022). This assumption is not valid for an evolving tissue like woven bone. In the woven bone, 6th-degree polynomial equations were proven to best fits the elastic modulus versus the ash fraction, predicted by a multiscale computational homogeneization model (García-Rodríguez and Martínez-Reina, 2017). In the *in silico* model, García-Rondríguez and Martínez-Reina (García-Rodríguez and Martínez-Reina, 2017) assumed isotropic orientation of collagen fibrils and used the ash fraction reported in the literature (Martínez-Reina et al., 2018). A simple exponential regression equation based on the ash fraction α , $E=12.88\cdot \alpha^{2.75}$ , was also provided with a significant correlation coefficient. To date, no previous work has proposed power laws based on experimental data to predict the apparent mechanical properties of woven bone at micro- or macro-scales considering the evolutionary structure of this tissue.

Various techniques have been developed to assess the bone inorganic matrix at macroscopic scale to measure bone mineral density or mineral content: computed tomography (Snyder and Schneider, 1991; Rho et al., 1995), micro-computed tomography, dual-energy X-ray absorptiometry (Hodgskinson and Currey, 1993; Bouxsein and Radloff, 1997), ashing the sample (Currey, 1988; Martínez-Reina et al., 2018) or weighing it (Schaffler and Burr, 1988; Martin and Boardman, 1993; Keller, 1994; Nicholson et al., 1997). To account for local variations in mineral content, scanning small angle X-ray scattering and wide angle X-ray scattering have been used to measure mineral crystal length and thickness (Roschger et al., 2020). Quantitative back-scattered electron microscopy is a validated method for determining spatially resolved calcium content (Roschger et al., 2020), and Fourier Transform infrared microspectroscopy and Raman microspectroscopy have been widely used to study the inorganic chemical compositional changes of the bone (Tarnowski et al., 2002; Akkus et al., 2004; McCreadie et al., 2006; Yerramshetty et al., 2006; Morris and Mandair, 2011), including mineralization, crystallinity, or carbonate substitution amongst others. For instance, investigating the inorganic chemical compositional changes during bone ageing using infrared and Raman microspectroscopy, it was concluded that Raman microspectroscopy is more sensitive for the inorganic matrix (Turunen et al., 2011). Bone healing processes have also been monitored with Raman spectroscopy (Uthgenant et al., 2007; Gamulin et al., 2013; Ahmed et al., 2018). Ahmed et al. (2018) studied early healing in calvarial defects, which heal spontaneously, with Raman Spectroscopy. They showed an increase in mineral/matrix and crystallinity ratios and a reduction in the carbonate/phosphate ratio after 14 days of healing. However, the long-term temporal evolution of the mechanical, chemical and morphological properties of woven bone during healing in critical-sized bone defects remains unknown. Beyond ash analysis, using Raman spectra or back-scattered electron signals as reliable input data for building power laws to predict the mechanical properties of bone tissue has not been explored to date.

Methods for evaluating the mechanical properties of bone tissue are more standardized. Macroscopic mechanical properties of cortical and trabecular bones have been traditionally evaluated through different mechanical testing including tension, compression, torsion, three-point bending, or buckling (Townsend et al., 1975; Ryan and Williams, 1989; Woo et al., 1991; Bayraktar et al., 2004; Beaupied et al., 2007). Local mechanics of mature tissue has been also assessed with ultrasound microscopy (Eriksen et al., 1994; Hengsberger et al., 2002; Bala et al., 2013), nanoindentation (Rho et al., 1995; 1999; Rho and Pharr, 1999; Zysset et al., 1999; Thurner, 2009) or atomic force microscopy (Asgari et al., 2019). However, for woven bone and its evolutionary properties, studies have used both *in vivo* (Dwyer et al., 1996; Aarnes et al., 2005; Mora-Macías et al., 2015a; b; Blázquez-Carmona et al., 2021a) and *ex vivo* approaches (Ohyama et al., 1994; Floerkemeier et al., 2010; Blázquez-Carmona et al., 2022) to provide values of local and apparent stiffness at different time-points during healing. Typically, *in vivo* mechanical properties are indirectly measured through instrumentation of surgically implanted fixations (Dwyer et al., 1996; Aarnes et al., 2005; Mora-Macías et al., 2015a; b; Blázquez-Carmona et al., 2021a). For *ex vivo* techniques, nanoindentation tests have proven valid to account for nanoscale heterogeneity and examine bone quality at a lower scale (Leong and Morgan, 2008; Manjubala et al., 2009; Mora-Macías et al., 2017; Mora-Macías et al., 2021). For instance, Manjubala et al. (2009) and Mora-Macías et al. (2017) measured the nanoindentation modulus for woven bone during fracture healing and bone transport processes, respectively.

*In silico* models are valuable for the understanding the course of healing from a mechanobiological perspective. Many researchers have sought to establish relationships between the mechanical properties of undifferentiated tissue and ultimate tissue phenotype formed in a wide variety of bone regeneration processes, such as fracture healing (Borgiani et al., 2017; Ghiasi et al., 2017), distraction osteogenesis (Isaksson et al., 2007; Reina-Romo et al., 2011; 2012) or tissue engineering (Byrne et al., 2007; Stops et al., 2010), amongst others. As a limitation, all these works develop phenomenological rules based on empirical observations and input parameters that are not sufficiently validated with experimental data, leaving room for improvement in accuracy. In particular, most of these models assumed constant values for the elastic modulus and porosity of the woven bone, regardless of its mineralization state or degree of matrix organization, which is not explicitly modeled. A constant porosity value of 80% and an elastic modulus of 1,000 MPa are typically assigned to the woven bone in most of these mechanobiological evolutionary models (Lacroix and Prendergast, 2002; Isaksson et al., 2007; Burke and Kelly, 2012).

The main aim of this study is to identify time-related changes at the macro- and micro-scales at the chemical, mechanical, and morphological properties of the woven bone during healing of a critical-size bone defect. There is a need for a better understanding of the woven bone micromechanics and the development of a reliable mechanical model of this tissue to improve the prediction of its stiffness. For this purpose, at the microscopic scale, micromechanical properties will be measured with nanoindentation; mineral and organic characteristics will be quantified through Raman spectroscopy; and structural features through micro-CT. At the macroscopic scale, ash and element analyses will be assessed together with a numerical model to reproduce the woven bone bulk mechanical behavior based on physical measurements. All these experimental data will be used to build power laws relating the elastic modulus with the structural and chemical properties of the woven bone for the first time.

## 2 Materials and methods

### 2.1 Origin and preparation of the woven bone samples

**FIGURE 1:**
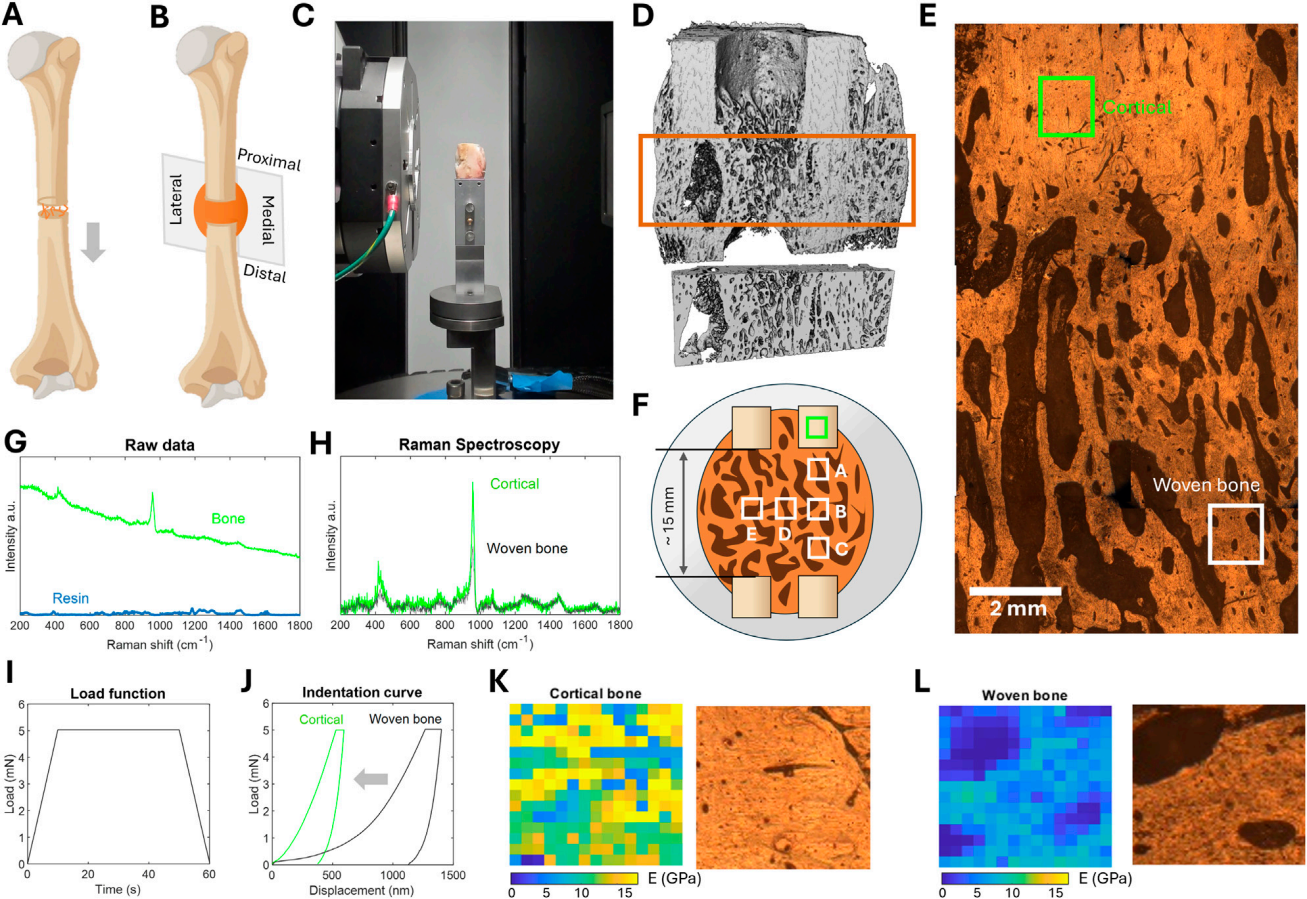
**(A)** Scheme of the osteotomized metatarsus and the distracion protocol, **(B)** scheme of the frontal plane sliced and analyzed from each operated limb, **(C)** image of a bone callus sample being imaged in the tomography, **(D)** 3D reconstruction of one of the bone calluses and volume of interest structurally analyzed, **(E)** visualization of microscope image of a bone callus sample. An example of cortical and woven bone areas to be analyzed by Raman Spectroscopy and nanoindentation are indicated, **(F)** scheme of the embedded bone callus samples and the areas analyzed, **(G)** example of a raw Raman spectra of the bone and the embedding resin, **(H)** comparison of corrected Raman spectra between cortical and woven bones, **(I)** load function applied by the indenter during a nanoindentation measure, **(J)** comparison of the indentation curve between cortical and woven bones, **(K)** nanoindentation of a cortical bone area, **(L)** nanoindentation of a woven bone area.

The woven bone samples analyzed in the current study originate from previous *in vivo* distraction osteogenesis experiments conducted on the right-back metatarsi of six skeletally-mature female Merino sheep (Blázquez-Carmona et al., 2021a; b). The animals followed a surgical intervention and a bone lengthening protocol approved by the Animal Ethics of the University of Córdoba (Reference 2021PI/21), in compliance with the European (2010/63/UE) and national (RD 1201/2005) regulations. During the surgery, an Ilizarov-type external fixator was implanted in the metatarsus, and a cross-sectional osteotomy was performed in an intermediate section of the diaphysis. Consequently, each metatarsus was divided into two initially unconnected bone fragments, with their alignment ensured by the fixator device. After a 15-day latency period during which the bone healing begins by way of a preliminary soft callus formation, the fragments were distracted at rate of 1 mm/day during 15 days (Figure 1A). The resulting 15-mm bone callus was then allowed to mineralize over time. The specimens were sacrificed at different time-points of the distraction and consolidation phases to analyze *ex vivo* different states of callus ossification: days 18, 29, 47, 64, 112 and 161 after surgery. More details on the design of the external fixator and the distraction protocol were provided in previous works (Blázquez-Carmona et al., 2020; 2021b). After sacrifice, operated limbs were immediately stored at −80°C.

Two slices of each bone callus, each approximately 3 mm thick, were cut in the frontal plane to address potential differences in the lateral-medial direction (Figure 1B). These cuts were performed using a Femi FM-785XL band saw (Femi, Castel Guelfo, Bologna, Italy) while the sample was freshly taken out of the freezer to maintain the integrity of both the surrounding soft tissues and the callus itself. The first slice was used for micro-CT, Raman Spectroscopy and nanoindentation analysis. For micro-CT, the sample was analyzed in the same condition as cut. However, the other techniques required embedding and subsequent surface polishing to achieve flat and parallel surfaces. For embedding, a 40-mm diameter cylindrical polypropylene mold (FixiForm, Struers, California, United States) and a slow-curing epoxy resin (Epofix, Struers, California, United States) were employed. This resin hardens at room temperature in 24 h, thus avoiding alterations in the mechanical or chemical properties of the sample due to high curing temperatures. Following a bone polishing guidelines provided in the literature (Mora-Macías et al., 2017), the surfaces of the embedded samples were polished by means of carbide papers (P600 to P4000) and diamond slurry (from 3 to 0.25 µm), cleaning them ultrasonically with distilled water after each polishing step. The second slice was used to analyze the chemical composition of each bone callus, for which the cortical fragments were excised. No additional preparation was needed for this second slice. After the slicing, samples were stored in PBS-soaked gauze and plastic wrap at −80°C until the testing day. They were thawed by placement in PBS at room temperature for 1 h prior to every test. Throughout the brief intervals between micro-CT, nanoindentation, and Raman spectroscopy tests, the samples were consistently kept hydrated in PBS-soaked gauze and plastic wrap at 4°C. As a reference for the interpretation and discussion of the micro-CT results, six trabecular and six cortical samples from the operated ovine metatarsus were also cut and preserved as detailed above. Similarly, as a control for the chemical analysis, cortical fragments of each operated metatarsus were prepared.

### 2.2 Micro-computed tomography of the bone calluses

Micro-CT images were taken using a Cougar tomography^®^ (Y.Cougar SMT, Yxlon, Hudson, Ohio, United States) with the following settings: ×4 optical magnification; exposure time of 9 s; voxel size of 3.72 µm); source setting of 50 kV and 90.02 µm); and a physical size of approximately 25 × 25 × 25 mm. An image of one of the samples in the micro-CT during imaging is provided for reference (Figure 1C). The tomographies were processed using the image analysis platform Thermo Scientific Avizo Software (Thermo Fisher Scientific, Waltham, Massachusetts, United States). A median filter (3D interpretation, 6 neighborhood pixels, 3 iterations) was initially applied to reduce the contrast, soften the edges of the woven bone, and facilitate segmentation. A mixed automatic-manually interactive thresholding was performed, followed by a small noise spot removal with a maximum size of 100 px. The 3D structures were cropped into an inner cube covering the entire bone callus volume. An example of a thresholded sample and the cropped bone callus volume of interest is provided (Figure 1D). From each 3D reconstructed callus, several parameters were determined: bone volume over total volume (BV/TV, module *Volume Fraction*), average trabecular thickness (Tb.Th, module *Average Object Thickness*), average trabecular separation (Tb.Sp, module *Average Space Thickness*), trabecular number (Tb.Nm, module *Average Object Number per slice*), degree of anisotropy (DA, module *Degree of Anisotropy*), and Structural Model Index (SMI, module *Structure Model Index*), which defines the relative prevalence of rods and plates in the structure.

The connectivity of the woven trabecular networks (Conn.D) was calculated using the Euler-Poincaré formula for a 3D structure X (Odgaard and Gundersen, 1993):

$$\chi \left(X\right)=\beta_{0}-\beta_{1}+\beta_{2}$$ (1)

where χ is Euler-Poincaré number measured using the software Avizo (module *Euler Number 3D*), $\beta_{0}$ is the number of objects, $\beta_{1}$ is the connectivity, and $\beta_{2}$ is the number of enclosed pores. Following the well-stablished procedure for cancellous bone (Odgaard and Gundersen, 1993), it was assummed trabecualae fully interconnected with no isolated solids $\left(\beta_{0}=1\right)$ and no marrow cavities within the bone $\left(\beta_{2}=0\right)$ . Note that the microporosities within the trabeculae will not be considered further as they are out of the analysis scale. Equation 1 may thus be reformulated as shown in Equation 2:

$$\beta_{1}=1-\chi \left(X\right)$$ (2)

Control trabecular and cortical bone samples (n = 6 per tissue type) were also measured and analyzed using micro-CT. However, BV/TV was the only parameter calculated in cortical samples due to the absence of trabecular structures in this tissue.

### 2.3 Material analysis using Raman Spectroscopy

For the spectroscopy and nanoindentation tests, six rectangular regions of interest (800 × 800 µm) were selected in both woven and cortical tissues from each of the embedded and polished bone callus samples. An example locations of a composition of images taken with the microscope to select these areas of interest is provided for reference (Figure 1E), highlighting examples of cortical and woven tissue. One area was always located in the proximal cortical fragment as a control reference (Figure 1F). The other five zones were positioned to cover the proximal-distal (A-B-C) and medial-distal (B-D-E) directions.

Raman Spectroscopy analysis was performed using a LabRAM Horiba Jobin Yvon (HORIBA, Kyoto, Japan) confocal Raman microscope. The embedded samples were periodically sprayed with PBS on their surface to maintain hydration. Spectra were collected with a 785 nm red laser at ×100 magnification, with a 5-s integration time and 20 accumulations. The spectral range was from 200 to 1800 ${cm}^{-1}$ . The microscope was calibrated against a silicon standard prior to imaging each sample. Examples of raw spectra taken by the Raman microscope are presented for reference (Figure 1G), showing bone (green) and background fluorescence mainly from the embedding resin (blue). Then, background fluorescence removal and a baseline correction were applied to each individual spectrum using a cubic spline interpolation. Eight point-spectra were taken at different locations within each selected zone, including the cortical one, resulting in a total of 48 spectra per bone callus sample. In the woven bone areas, all spectra were taken in areas within the trabecula, thus avoiding porous points. An overview of the differences between corrected spectra from the cortical region and those from a woven bone area is also provided (Figure 1H). The bands to analyze bone mineral phase are the phosphate band at ∼ 959 ${cm}^{-1}$ ( $V_{1}$ ${PO4}^{3-}$ ), ∼ 422 ${cm}^{-1}$ ( $v_{2}$ ${PO4}^{3-}$ ) and the carbonate band at ∼ 1070 ${cm}^{-1}$ (Mandair and Morris, 2015; Fraulob et al., 2020). The full-width half-height (FWHM) of the $v_{1}$ ${PO4}^{3-}$ band is also interesting due to its inversely proportionality to mineral crystalline length and, therefore, is an indirect measure of mineral crystallinity. The most widely used bands to measure the matrix content are Proline at ∼ 853 ${cm}^{-1}$ , Hydroxyproline at ∼ 872 ${cm}^{-1}$ , Amide III at ∼ 1250 ${cm}^{-1}$ , and Tyrosine at ∼ 1607 ${cm}^{-1}$ (Mandair and Morris, 2015; Fraulob et al., 2020). The spectra were processed in MATLAB (R2022a, MathWorks, Natick, Massachusetts, United States) to calculate intensity peaks (I), areas under the peaks (A), and broadness as full width at half maximum (FWHM). The parameters and ratios calculated are detailed in Table 1, indicating if they are related to the mineral or oganic phase.

**TABLE 1:** Raman parameters in cortical and woven bone tissues calculated for each Raman spectra. I: maximum peak intensity, A: area under the peaks, FWHM: full width at half maximum. the subscript indicates the Raman shift position ( ${cm}^{-1}$ ).

Raman paramenter	Tissue phase	Ratio
Crystallinity	Mineral	$\frac{1}{FWHM_{959}}$
Carbonate-to-phosphate	Mineral	$\frac{I_{1070}}{I_{959}}$
$v_{1}$ PO $4^{3-}$ /Proline mineral-to-matrix	Mineral/Organic	$\frac{I_{959}}{I_{853}}$
$v_{1}$ PO $4^{3-}$ /Tyrosine mineral-to-matrix	Mineral/Organic	$\frac{I_{959}}{I_{1607}}$
$v_{2}$ PO $4^{3-}$ /Amide III mineral-to-matrix	Mineral/Organic	$\frac{A_{422}}{A_{1250}}$
Carbonate-to-matrix	Mineral/Organic	$\frac{A_{1070}}{A_{\mathrm{1620-1700}}}$
Hydroxyproline-to-proline	Organic	$\frac{I_{872}}{I_{853}}$
Amide I-to-Amide III	Organic	$\frac{I_{\mathrm{1620-1700}}}{I_{1250}}$

### 2.4 Mechanical properties using nanoindentation

The elastic modulus of the tissue was determined by nanoindentation tests on the same regions of interest described in section 2.3, including the cortical zones. Nanoindentation was performed using a Nanotest indenter (Nanotest, Micro Materials Ltd. Wrexham, United Kingdom) equipped with a Berkovich diamond indenter. In each area, 16 × 16 indentations were made, spaced 50 µm apart in both directions, corresponding to the minimum trabecula thickness of naïve woven bone tissue (López-Pliego et al., 2016). Thus, a total of 256 indentations were performed per area, covering the entire region of interest. Samples were sprayed with PBS from time to time to preserve their hydration. The load applied to each indentation point was increased at a rate of 0.5 mN/s to a maximum of 5 mN (Figure 1I). Once the maximum load was reached in approximately 10 s, it was held constant for 40 s. The unloading was performed at the same rate. The indentation depth, or the indenter’s displacement, is a variable parameter that depends on the hardness of the tissue, being lower in cortical bone (Figure 1J). The Oliver and Pharr method was used to calculate the elastic modulus (E) from the load-depth data (Oliver and Pharr, 1992). Examples of nanoindentation maps measured on cortical and woven bone regions are provided (Figures 1K, L, zones of interest marked in Figure 1E). The average elastic modulus per zone and its standard deviation were calculated by eliminating from the calculation those areas of porosity identified by microscope images or with elastic modulus less than 2 GPa.

### 2.5 Chemical analysis of the tissue

The second slice was employed for ash analysis and elemental analysis. The samples were initially ground with a sterilized pestle and mortar. They were then immediately dried in a BINDER VD 23 vacuum drying chamber (BINDER GmbH, Tuttlingen, Germany) using heating cycles at 105 ± 2°C for 1 h each, measuring the samples’ weight at the end of each cycle until it stabilized. The resulting weight combines that corresponding to the mineral and organic phases $\left(m_{m}+m_{o}\right)$ of the woven bone tissue after removing water. To obtain the ash fraction, the samples were ashed in a Nabertherm Muffle Furnace (Nabertherm GmbH, Lilienthal Germany) following a protocol published in the literature (Martínez-Reina et al., 2018): (1) the temperature was initially increased from room temperature to 250°C over 30 min, and held at this temperature for 1 h; (2) the temperature was gradually increased to 650°C over 30 min, and held for 2 h; (3) the sample was then taken out of furnace and weighed; (4) the sample was reintroduced into the furnace at 650°C and maintained at this temperature for 30 min. Steps 3 and 4 were repeated until a constant weight was achieved. This process allowed removing the organic material. Therefore, the weight obtained at the end of the protocol corresponds to the mineral phase exclusively $\left(m_{m}\right)$ . The ash fraction α was finally calculated using Equation 3.

$$\alpha =\frac{m_{m}}{m_{m}+m_{o}}$$ (3)

An elemental analysis was also performed on the ashed samples to measure the calcium (Ca) mass percentage and trace element substitutions, mainly stable isotopes of carbon with oxygen in the carbonate ( ${CO}_{3}^{2-}$ ) and hydrogen phosphate ( ${HPO}_{4}^{2-}$ ) (Martínez-Reina et al., 2018; Katzenberg, 2020). Hence, the mass percentages of carbon (C) and phosphorus (P) were evaluated as an indirect measure of these impurities. Other possible calcium substitutes measured in the current study were sodium (Na), potassium (K) and other alkaline earth elements similar to calcium, including magnesium (Mg) or strontium (Sr) (Bergstrom and Wallace, 1954; Katzenberg, 2020).

The elemental analysis of the carbon content was conducted using a TruSpec Micro analyzer (LECO Corporation, Michigan, United States). The calcium and phosphorus contents were measured with an inductively coupled plasma optical emission spectrometer SpectroBLUE (Spectro Analytical Instruments, Kleve, Germany) after the samples were dissolved in hydrochloric acid. As stated in section 2.1, one cortical slice from each metatarsal diaphysis was also prepared and analyzed using the methodology detailed above (six cortical fragments in total).

### 2.6 Definition of power laws for woven bone

The *ex vivo* experiments described in the previous sections will elucidate the evolution of the mechanical and chemical properties of woven bone during healing at the tissue scale, as well as the structural changes at the apparent level towards a cortical microarchitecture. In the literature, ash fraction or calcium content have been shown to be effective predictors of cancellous and cortical bone’s mechanical properties (Currey, 1988; Hernandez et al., 2001; García-Rodríguez and Martínez-Reina, 2017). Therefore, these parameters were used to define single-parameter powers laws that predict the woven bone elastic modulus over the regeneration time, following Equation 4:

$$E=a\cdot x^{b}$$ (4)

where E is the elastic modulus, a are b are empirical constants derived from experimental data, and the variable x represents the predictor parameter. Specifically, empirical constants were fitted using the mean elastic modulus per sample measured by nanoindentation, evaluating the following experimental parameters as a predictors: ash fraction (x=α) , calcium content ( x= Ca) and phosphorus contents ( x= P) measured by chemical analysis, and Raman ratios, $v_{2}$ ${PO4}^{3-}$ /Amide III mineral-to-matrix $\left(x=A_{422}/A_{1250}\right)$ and $v_{1}$ ${PO4}^{3-}$ /Tyrosine mineral-to-matrix $\left(x=I_{959}/I_{1607}\right)$ . Note that the $v_{2}$ ${PO4}^{3-}$ /Amide III ratio has been extensively shown to be proportional to calcium content, as measured by quantitative backscattered electron microscopy (Roschger et al., 2014; Mandair and Morris, 2015). The phosphate $v_{1}$ ${PO4}^{3-}$ , as a ratio indicative of organic matrix content, is also widely accepted as a measure of mineral content.

Given that the experimental data used to fit the empirical coefficients were measured at the micro-scale, the previous models predict the elastic modulus at the tissue scale. Consequently, a two-parameter power law function was also proposed in Equation 5 to differentiate the influence of bone volume (BV/TV) and ash fraction (α) on an apparent macro-scale elastic modulus, as has been previously applied in cortical bone studies (Currey, 1988; Hernandez et al., 2001):

$$E^{*}=a\cdot \alpha^{b}\cdot {\left(BV/TV\right)}^{c}$$ (5)

To adjust the coefficients of this second model, the volume fraction (BV/TV) measured by micro-CT were employed. Moreover, the apparent elastic modulus was taken by the model provided by Blázquez-Carmona et al. (2021a) and shown in Equation 6, which is based on experimental gait force data measured *in vivo* through instrumented external fixators and a load platform during gait tests on the same animals whose bone calluses were used in this work (R-square = 0.933, *p*-value < 0.001):

$$E^{*}=3.76\cdot e^{0.048\cdot t}$$ (6)

where t is the day after surgery, and $E^{*}$ is the apparent elastic modulus in MPa. Evaluating the time-points analyzed in this study, the apparent elastic modulus ranges between 0.01–8.53 GPa. The determination coefficients, R-square and *p*-values, were also calculated for all power laws defined above.

## 3 Results

### 3.1 Evolution of the woven bone microarchitecture

**FIGURE 2:**
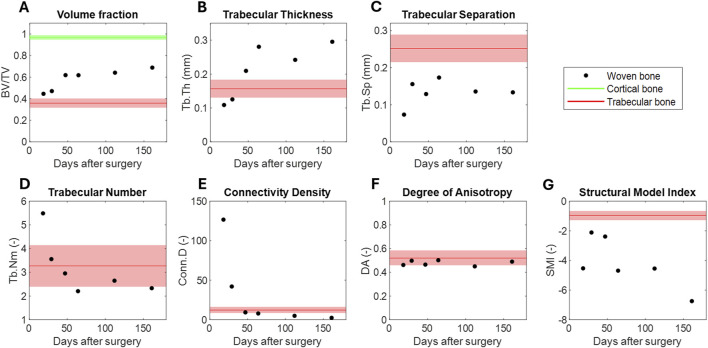
Evolution of the woven bone’s microarchiture measured from micro-tomographic images: **(A)** volume fraction, **(B)** trabecular thickness, **(C)** trabecular separation, **(D)** trabecular number, **(E)** connectivity density, **(F)** degree of anisotropy, **(G)** structural model index. The average value and standard deviation of the cortical and trabecular data is provided as a reference in green and red, respectively.

The evolution of the structural parameters in the 3D reconstructed woven bone calluses were measured from the micro-tomographic images (Figure 2). The BV/TV raised during the mineralization process (Figure 2A). The calluses were partially mineralized shortly after the surgery, comprising 44.59 % of the total callus volume after 18 days. Bone volume increased slightly over time, reaching 68.89 % after 161 days. Throughout the analyzed period, the calluses had a volumetric fraction above that of the spongy bone in the proximal epiphysis (35.89 ± 4.42%, red data) but below the surrounding cortical tissue (96.80 ± 1.52%, green data). The size of the trabeculae increased substantially during the regeneration period from approximately 0.11 mm, similar to that of trabecular bone, to 0.29 mm (Figure 2B). In contrast, trabecular separation did not show a specific evolution, consistently remaining below the separation in the proximal cancellous bone (Figure 2C). Both the trabecular number Tb. Nm (Figure 2D) and the connectivity Conn. D (Figure 2E) exhibited a similar pattern. A high number of trabecular structural structures with high connectivity were observed shortly after surgery. However, as trabeculae increased in size, these parameters decreased rapidly and stabilized at similar values to trabecular bone values. The degree of anisotropy (DA) remained constant between 0.45-0.51 (Figure 2F). Thus, similar to trabecular bone, although the structures showed a certain preferred orientation during trabecular formation, they were neither perfectly isotropic (DA = 0) nor completely anisotropic (DA = 1). Finally, the structural model index (SMI) negatively increase, indicating an increase in the concavity of the trabecular surfaces (Figure 2G).

### 3.2 Elastic modulus of the bone callus

**FIGURE 3:**
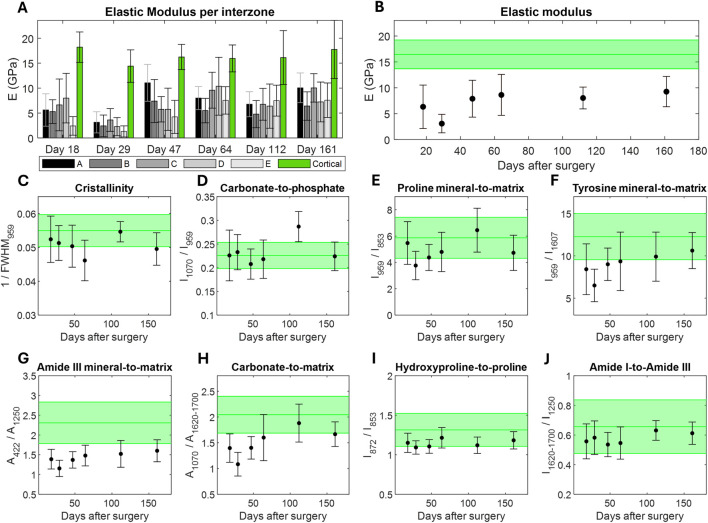
Evolution of the micro-scale woven bone’s elastic modulus and chemical composition measured by nanoindentation and Raman Spectroscopy, respectively. Mechanical properties: **(A)** elastic modulus by callus zones in each of the samples analyzed, **(B)** average elastic modulus of all the indentations made in all analyzed areas of the sample. Chemical structure: **(C)** Cristallinity, **(D)** Carbonate-to-phosphate, **(E)** Proline mineral-to-matrix, **(F)** Tyrosine mineral-to-matrix, **(G)** Amide III mineral-to-matrix, **(H)** Carbonate-to-matrix, **(I)** Hydroxyproline-to-proline, **(J)** Amide I-to-Amide III. Data from cortical bone (mean and standard deviation) is provided in green.

The average and standard deviation of the elastic modulus were calculated in each analyzed area, including the cortical region (Figure 3A). Additionally, nanoindentation maps of the analyzed zones, along with their corresponding microscopic reference images, are provided in the Supplementary Material (Supplementary Figure S1). Overall, no remarkable differences were found between the areas in any analyzed direction of the callus, either the proximal-distal direction (A-B-C) or the medial-lateral direction (B-D-E). In all samples, the elastic modulus of cortical tissue is double or triple that of woven bone. The temporal evolution of the elastic modulus of the samples was also analyzed as the global average of all regions of interest (Figure 3B). Note that the control cortical data provided in Figure 3B encompasses the mean and standard deviation from all cortical nanoindentations across all samples/animals (256 cortical indentations per sample, 1,536 in total). Initially, the elastic modulus ranged from 3.07 to 6.32 GPa in the first weeks after surgery, and it increased slightly to 9.25 GPa after 6 months. During the analyzed period, the mechanical properties of woven bone remained notably lower than those of cortical bone, whose elastic modulus was 16.44 ± 2.79 GPa.

### 3.3 Chemical composition of the woven bone

In this study, the chemical structures of woven and cortical bones were analyzed on both micro- and macro-scales. On the micro-scale, the evolution of the Raman parameters were calculated for each woven bone sample (Figure 3). The data is provided as the average ratios of all zones and all spectra taken from each. The control cortical data provides the average ratios of all spectra measured in all samples (6 spectra per sample, 36 in total). The results per areas of each sample is also provided in the Supplementary Material (Supplementary Figure S2). In both figures, the ratios calculated for the reference cortical tissue are shown in green. The naïve woven bone tissue exhibited a degree of crystallinity comparable to that of cortical tissue from few days after surgery, without appreciable evolution over time (Figure 3C). During regeneration, the carbonate-to-phosphate ratio was also similar to that of cortical bone, without noteworthy differences in their standard deviation (Figure 3D). The mineral amount of Phosphate v1 and v2 was normalized by the organic components of the matrix: Proline (Figure 3E), Tyrosine (Figure 3F), and Amide III (Figure 3G), respectively. Although no trend was observed when normalizing by Proline compared to the cortical tissue, the ratios against the other organic phases began notably lower in woven bone compared to mature bone tissue, tending to approach cortical values during the regeneration phase. The carbonate content of the newly formed bone tissue also increased relative to the organic phase until reaching cortical values (Figure 3H). No trend was found in the Hydroxyproline-to-Proline (Figure 3I) and Amide I-to-Amide III (Figure 3J) ratios, but their standard deviations were lower than those calculated for the same ratios in cortical bone.

**FIGURE 4:**
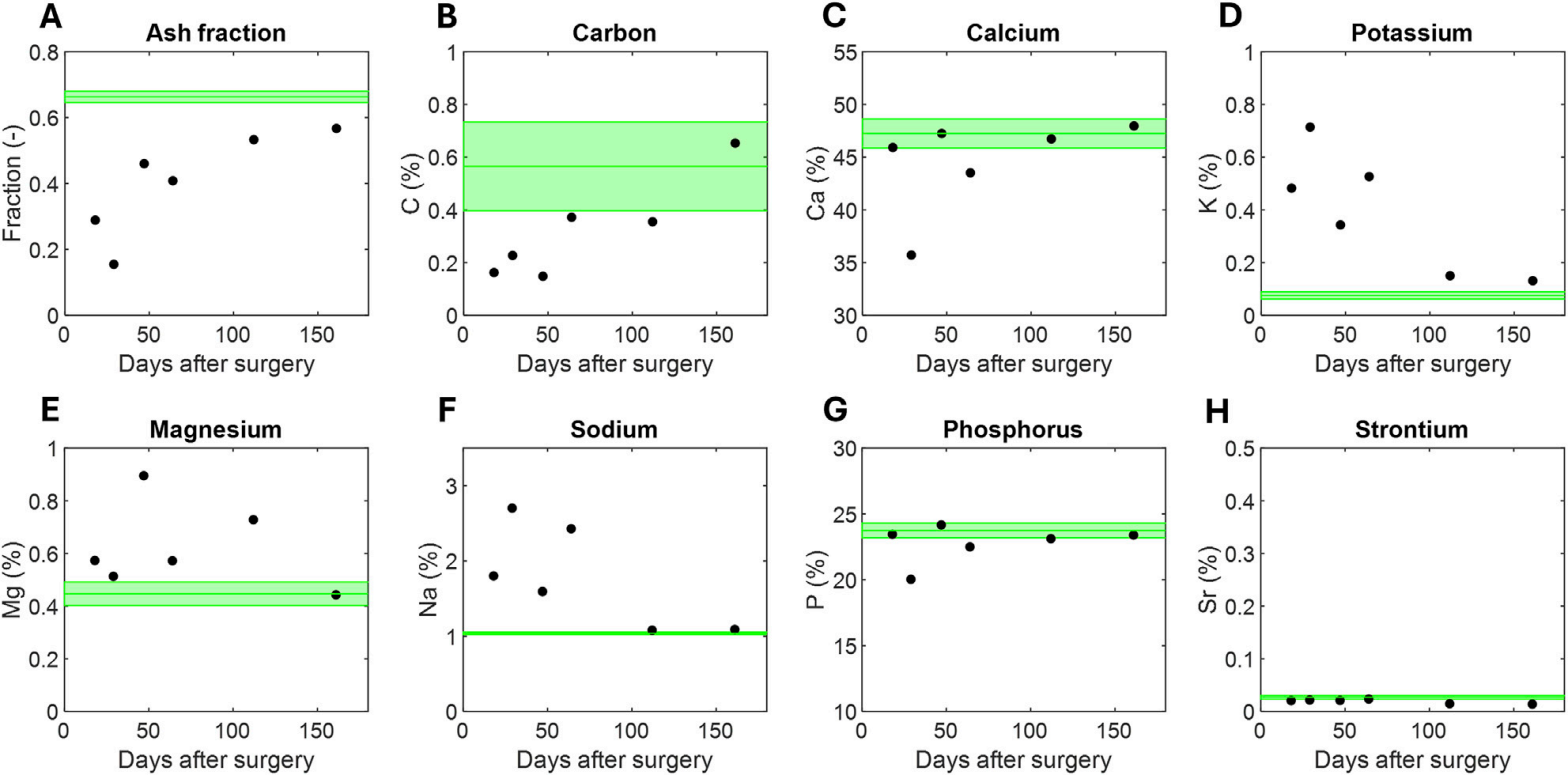
Evolution of the macro-scale woven bone’s chemical composition measured with micro-analysis: **(A)** ash fraction, **(B)** carbon percentage, **(C)** calcium percentage, **(D)** potassium percentage, **(E)** magnesium percentage, **(F)** sodium percentage, **(G)** phosphorus percentage, **(H)** strontium percentage. Cortical data (mean and standard deviation) is provided as a reference in green lines.

Regarding the macro-scale chemical composition, the evolution of the ash fraction over the regeneration time was investigated (Figure 4A). The results obtained from the six control cortical fragments are also provided as a mean and standard deviation (green data). The mass fraction of the mineral component versus the organic component of the tissue increased as the collagen template mineralized over time. Consequently, the ash fraction increased progressively from 0.15 to 0.29 in the less mature calluses to 0.57 in the most ossified ones. This more consolidated callus reached an ash fraction similar to that of cortical tissue, 0.66 ± 0.01 % .

The micro-elemental determinations of the chemical components specified in section 2.5 are also provided (Figures 4B–H). The carbon content (C % ) slightly increased from 0.20 to 0.65 % . However, the percentage of this element in all samples, including their cortical areas, remained relatively low ( <1% ). Calcium (Ca % ) was the predominant element in all samples, with percentages ranging from 35.71 to 47.96 % (Figure 4B). Note that the calcium content in a cortical bone sample was estimated at 47.25 ± 1.38 % . Although a slight increase is seen throughout the regeneration phase, some of the least mature samples already showed calcium levels similar to cortical levels. Potassium (K % , Figure 4D), magnesium (Mg % , Figure 4E), and sodium (Na % , Figure 4F) followed a similar trend. All of these impurities began with percentage contents (0.48-0.71 % for K; 0.57-0.89 % for Mg; 1.80-2.71 % for Na) above the cortical values of 0.08 ± 0.01 % , 0.45 ± 0.04 % , and 1.04 ± 0.01 % , respectively. However, all of them normalized over the period analyzed. Phosphorus (P % ) was the second most abundant element (Figure 4G), maintaining a relatively constant percentage between 20.03 and 24.16 % , similar to that of cortical bone (23.74 ± 0.55 % ). Finally, the percentage of strontium (Sr % ) was negligible in all samples, remaining around 0.02 % (Figure 4H).

### 3.4 Power laws: influence of structural and chemical properties of the elastic modulus

**FIGURE 5:**
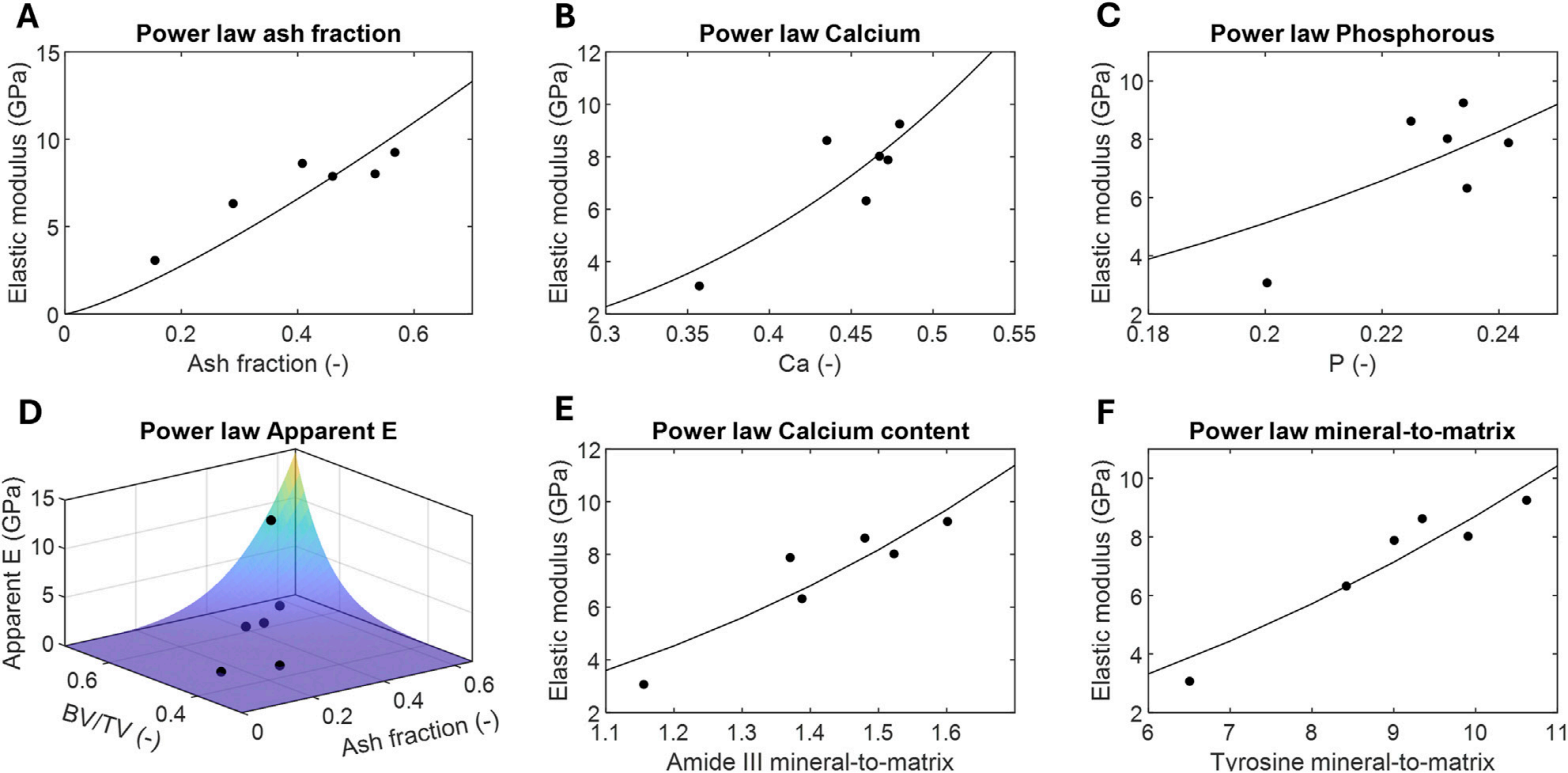
Correlations of the power laws defined in Table 2 to predict the tissue or apparent elastic modulus from the following structural and chemical parameters measured *ex vivo*: **(A)** ash fraction from chemical analysis, **(B)** calcium content from chemical analysis, **(C)** phosphorus content from chemical analysis, **(D)** ash fraction from chemical analysis and volume fraction from micro-CT, **(E)** $v_{2}$ PO $4^{3-}$ /Amide III mineral-to-matrix from Raman Spectroscopy, undertood as the calcium content, **(F)** $v_{1}$ PO $4^{3-}$ /Tyrosine mineral-to-matrix from Raman Spectroscopy.

Table 2 presents the different power laws, the value of the empirical constants after adjustment with the experimental data, as well as the goodness-of-fit parameters: the R-square and the *p*-value. Additionally, these best-fit power law models of the tissue and apparent elastic modulus was also graphically represented as functions of the predictor parameters, plotted against the experimental data (Figure 5). All single-parameter adjustments demonstrated high predictive strength of the tissue elastic modulus, with all R-square values exceeding 0.5 and most of *p*-values below 0.05. The highest degrees of significance were observed in correlation based on Raman parameters. The largest resulting predictor exponent values were found in the Calcium content measured by chemical analysis (b =2.86 ) and by Raman spectroscopy (Amide III mineral-to-matrix, b =2.64 ).

**TABLE 2:** Definition of the power laws of the tissue and apparent elastic modulus ( E in GPa) based of different structural and chemical measured parameters, fitting constants, and goodness-of-fit parameters, R-square and *p*-values. Note that Calcium (Ca) and Phosphorus (P) in the power laws are expressed on a per unit basis. *Apparent elastic modulus measured *in vivo* by Blázquez-Carmona et al. (Blázquez-Carmona et al., 2021a).

Parameter	Power law	Constants			R-square	*p*-value
		a	b	c		
Ash fraction	$E=a\cdot \alpha^{b}$	20.84	1.26	—	0.55	0.091
Calcium	$E=a\cdot Ca^{b}$	71.56	2.86	—	0.72	0.031
Phosphorus	$E=a\cdot P^{b}$	349.85	2.62	—	0.56	0.104
Ash and volume fraction	$E^{*}=a\cdot \alpha^{b}\cdot BV/TV^{c}$	2.74e3	3.88	9.98	0.88	0.005
Amide III mineral-to-matrix	$E=a\cdot {\left(A_{422}/A_{1250}\right)}^{b}$	2.79	2.64	—	0.83	0.011
Tyrosine mineral-to-matrix	$E=a\cdot {\left(I_{1607}/I_{853}\right)}^{b}$	0.12	1.89	—	0.90	0.003

The two-parameter power law model based on the ash fraction and bone volume fraction measured in the current study, alongside the macro-scale apparent elastic modulus provided by Blázquez-Carmona et al. (2021a) (Figure 5D), was also found to be highly significant (R-square = 0.88 and *p*-value = 0.005). Furthermore, the best-fit volume fraction exponent value was nearly 3-fold the ash fraction exponent value, at 9.98 versus 3.88.

## 4 Discussion

Quantifying the structural, chemical, and mechanical properties of the woven bone is crucial for understanding the mechanobiology behind bone regeneration and assessing the effectiveness of ortophaedic approaches for healing critical-sized bone defects. Equally important is the parallel development of numerical models that optimize key engineering parameters to control this biological process and prevent non-unions. There is a notable lack of micromechanical models of woven bone in the literature based on *ex vivo* experimental data that consider changes in tissue properties at different regeneration phases. The multiscale research presented here analyzed the evolution of the trabeculae organization in this immature tissue through micro-CT, its elastic modulus using nanoindentation, its mineral quality via Raman spectroscopy, and its chemical composition with ash analysis. From these experiments, power laws based on physical measurements were defined for the first time, relating the elastic modulus of the woven bone with its porosity and mineralization state. In addition, the data and power laws provided in this study are highly extrapolated to human bone tissues due to the ovine nature of the samples. High similarities in bone composition were reported between mature sheep and humans in the literature (Newman et al., 1995). Similarly, comparable metabolic and bone remodeling rates were found using dual energy X-ray absorptiometry (den Boer et al., 1999). The results obtained in this study also try to address some open questions in the field: Why is the stiffness of woven bone lower than that of cortical bone? Is it solely due to the mineral content, or is it influenced by the structural factors as well? Do structural and mineral changes equally contribute to the bulk mechanical properties of woven bone? Are Raman spectra valid inputs for defining power laws?

The mechanical properties of the woven bone tissue exhibited a slight increase over the analyzed regeneration period, as indicated by the elastic modulus measured by nanoindentation, which ranged from 3.07 to 9.25 GPa (Figures 3A, B; Supplementary Figure S1). However, the stiffness levels characteristic of cortical tissue, approximately 16.44 ± 2.79 GPa, were not reached even after 161 days post-surgery. Previous studies also used nanoindentation to measure the elastic modulus of bone calluses formed during other regeneration processes (Leong and Morgan, 2008; Manjubala et al., 2009; Mora-Macías et al., 2017). Manjubala et al. (2009) reported values in the range of 2–13 GPa over the first 9 weeks of a fracture healing experiment in an ovine tibia. Thus, the woven tissue associated to this regeneration process reached higher stiffness at a greater speed, likely due to the smaller size of the bone gap in fracture healing, approximately 3 mm in this study. Similarly, Leong and Morgan (Leong and Morgan, 2008) conducted indentation tests on rodent fracture healing calluses, reporting an elastic modulus ranging from 0.03 to 1.01 GPa at day 35 after fracture. These results are considerably lower than those observed in the current study, possibly due to the different animal model used and the earlier healing stage analyzed. In another study, Mora-Macías et al. (2017) also measured elastic modulus values ranging from 7 to 14 GPa in bone transport calluses of the ovine metatarsus with the same critical size of 15 mm. Specifically, they reported an elastic modulus of 11 GPa after 161 days in bone transport (Mora-Macías et al., 2017), closely matching the 9.25 ± 2.93 GPa measured in this study during bone lengthening at the same time-point. The possible lower degree of mechanical maturation in the bone lengthening callus could be explained by a lower mechanical stimulation associated to the less mobility and bearing capacity of the specimens after the indirect elongation of the surrounding soft tissues (Blázquez-Carmona et al., 2021b). These findings reflect a slower recovery of the mechanical properties of the woven bone at the tissue scale throughout its remodeling phase. It is important to highlight that nanoindentation tests specifically excluded the influence of inter-trabecular macroporosities on the average data provided. Consequently, the observed lower stiffness can only be attributed to the presence of microporosities or other microstructural features at smaller length scales, or to differences in the chemical composition and organization of the deposited mineral phase, as discussed later.

The mineral content in woven bone has been previously examinated in the literature. Manjubala et al. (2009) measured calcium content directly from back-scattered electron microscopic images (2D mineral content), showing inhomogeneity in the callus mineralization with a maximum Ca content of 20% of Ca that saturates at week 9 after surgery. In cortical bone, Turunen et al. (2011) investigated the temporal evolution of mineral content during aging with Infrared and Raman spectroscopy. They found that while mineralization increases over time, hydroxyapatite crystals mature more slowly. Ahmed et al. (2018) reported an increase in the mineral-to-matrix and crystallinity ratios during the first 14 days of healing in calvarial defects through Raman Spectroscopy. In our study, bone mineralization during healing was quantified at the microscopic scale using several Raman parameters, including mineral-to-matrix and carbonate-to-matrix ratios. Figure 3 demonstrates the positive correlation between the Tyrosine and Amide III mineral-to-matrix and healing time, reaching values comparable to those of cortical bone at day 161. These measurements provide a quantitative assessment of the extent of bone mineralization, directly related to ash weight (Gourion-Arsiquaud et al., 2009). Crystallinity, which is a measure of the crystal size development (Turunen et al., 2011), remained nearly constant during the consolidation phase, with values similar to those of the cortical bone (Figure 3C). Given that osteoid is deposited by osteoblasts at different locations and timing within the callus, this finding suggests that the maximum crystal size is obtained in an early stage of regeneration. Despite the increase in the carbonate-to-matrix ratio (Figure 3H), the carbonate-to-phosphate ratio (Figure 3D) showed no trend during the consolidation phase, maintaining values akin to those of cortical bone. Similar results were reported in calvarian healing (Ahmed et al., 2018). This ratio represents type-B carbonate substitution into hydroxyapatite, a process not fully understood but associated with increased solubility and decreased mechanical performance of the tissue (Pan and Darvell, 2010). Literature suggests this substitution is more related to aging, as carbonate peak areas and intensities increase with age (Boskey and Robey, 2013; Turunen et al., 2013). Regarding the hydroxyproline-to-proline ratio, the literature proved that it negatively correlates with the maturation of the tissue (Shah et al., 2019). However, it keeps constant in woven bone and within the standard deviation range of cortical bone (Figure 3G). A similar trend was observed for the Amide I-to-Amide III ratio, which remained approximately constant above 1 (Figure 3J). It follows a very similar trend to crystallinity, as both processes are closely correlated with the bone formation. Initially, naïve collagen fibers are created followed by an enzymatically crosslinking (Knott and Bailey, 1998). These fibers then mineralized and served as scaffolds for the nucleation and growth of the additional mineral crystals (Gourion-Arsiquaud et al., 2009). A high Amide I-to-Amide III ratio indicates a predominance of functional and mature collagen crosslinks, whereas a predominance of Amide III suggests high activity of nonfunctional precursor collagen with reducible crosslinks (Coelho et al., 2014; Mandair and Morris, 2015; Fraulob et al., 2020). Thus, woven bone exhibits an increase in procollagen molecules and immature crosslinks during the analyzed regeneration phase.

Figure 4 also reported the chemical analysis performed by ash analysis and elemental study. The ash fraction, phosphorous and calcium content increased nonlinearly with healing time with a plateau nearly the cortical reference values. Phosphorous and calcium contents are also in line with the infrared and X-ray analysis on natural hydroxyapatite (Janicki et al., 2012). Numerous substitutors were reported in hydroxyapatite crystals in the literature, including carbon, potassium, magnesium, sodium, or strontium (Bergstrom and Wallace, 1954; Buddhachat et al., 2016; Katzenberg, 2020). For potassium and sodium, the decrease with time was quantified (Figures 4D, F). However, none of the above substitutes seems to play a key role during the bone regeneration process.

Regarding the structural features of the woven bone at the trabecular scale, as measured by micro-CT, the BV/TV increased up to 0.69. This increase is primarily due to an increased in the trabecuale thickness (Tb.Th) as the different mineralization fronts advance (Figure 2). Previous studies have already examined bone volume fraction using different techniques and length scales. For instance, Blázquez-Carmona et al. (2021a) and Mora-Macías et al. (2021) measured the bone volume fraction from CT macroscopic images in bone lengthening and bone transport, respectively. In these studies, the volume fraction ranged from 88.18% to 93.97% in bone lengthening and 87.55%–100% in bone transport at days 121–161 after surgery. The lower values in our study are more consistent with estimates reported in bone transport calluses (Mora-Macías et al., 2021), which used direct segmentation of 2D micrographs based on x-ray grey-scale images. Thus, the porosity at the trabecular scale appears to significantly impact the estimation of the bulk volume fraction. This structural feature likely contributes to the differences found in the same period of time between the micro-scale elastic modulus measured in this study (3.07–9.25 GPa), and the lower apparent elastic modulus measured *in vivo* by Blázquez-Carmona et al. (2021a) (0.01–8.53 GPa), input in a later discussed power law. Compared to the trabecular tissue, woven bone only exhibits BV/TV levels similar to those observed in this study or those reported in femoral bone at the early stages of regeneration (Mittra et al., 2005; Teo et al., 2007). The other three-dimensional microarchitectural parameters of woven bone (Figure 2) could not be directly compared to other regenerating tissues due to the lack of reported data in the literature, to our knowledge. Therefore, comparisons are limited to cancellous bone tissue data from this study or literature sources (Mittra et al., 2005; Teo et al., 2007; Turunen et al., 2013). The aforementioned increase in Tb.Th above cancellous values occurs alongside a progressive reduction of the number (Tb.Nm) and interconnectivity (Conn.D) of woven trabeculae, more similar to that of healthy metatarsal trabecular tissue, 3.26 ± 0.87 ${cm}^{-1}$ and 12.25 ± 3.96, respectively. These reference trabecular values are slightly higher than those of healthy ovine or human femoral trabecular tissue reported in the literature (Mittra et al., 2005; Teo et al., 2007; Turunen et al., 2013), probably due to physiological differences between bones. The stabilization of the woven trabecular separation around 0.13–0.17 mm (Figure 2C) contrasts with the constant increase in Tb.Th, which can only be explained by the remodeling of already mineralized tissue in the medullary cavity or the outer interzones of the callus farthest from the original cortical bone. The degree of anisotropy (DA) remained similar to that of trabecular tissue in the same bone (Figure 2F) or to values reported in human femoral bone by Turunen et al. (2013), Finally, SMI evolved negatively from around 0, typical for trabecular bone (Mittra et al., 2005; Turunen et al., 2013), indicating a transition from a rod-and-plate structure typical of trabecular bone to a denser structure characterized by void spaces (Hildebrand and Rüegsegger, 1997). These results highlight the dynamic evolution of woven bone microstructure, from numerous disorganized thin trabeculae at the beginning of the consolidation phase to a structure with fewer but thicker and better-interconnected trabeculae.

The previous experimental data facilitated the definition of the power laws presented in Table 2, which serve as valuable tools for predicting the elastic modulus of woven bone in future *in silico* models at both macro- and micro-scales. The varied laws allow for the assignment of woven bone tissue mechanical properties based on different experimental results, not necessarily mechanical testing. Except for the current study, woven bone correlations have never been established using mechanical, structural and chemical parameters measured directly from *ex vivo* samples. The exponents of these expressions ( b and c fitting constants in Table 2) provide insights into the degree of influence of chemical composition (measured at the macro- scale by ash and elemental analyses and at the micro-scale by Raman spectroscopy) and microstructural morphology on the mechanical properties of the woven bone at both macro- and micro-scale. For instance, the ash fraction appears to have a lower relative influence on the elastic modulus measured by nanoindentation (b=1.26) over a broad range of ash fraction values compared to the calcium or phosphorous contents (b=2.62−2.86) . This exponent is also slightly lower than those in equivalent power laws reported in the literature (Schaffler and Burr, 1988; Currey, 1988; Hernandez et al., 2001). The differences can be attributed to the use of compact and cancellous bone tissues from different mammalian species, the range of ash fractions analyzed, and the different loading conditions in the mechanical characterization. The degree of significance of all correlation (R-square in range of 0.55–0.90 and most of *p*-values below 0.05) is consistent with previous works (Schaffler and Burr, 1988; Currey, 1988; Hernandez et al., 2001). However, Raman parameters, Amide III mineral-to-matrix and Tyrosine mineral-to-matrix, were found to correlate more significantly with elastic modulus than ash, calcium or phosphorus fractions (Figures 3A–C, E, J; Table 2). We hypothesize it could also be due to significant chemical heterogeneity in the tissue. Note that the Raman spectra were taken in the same areas of woven bone as those measured by nanoindentation. We also define a two-parameter function to explain more of the variance in the bulk mechanical properties of the woven bone than single-parameter functions based solely on chemical factors. Thus, the combination of ash fraction with the evolution of the BV/TV provides a significant correlation with the apparent elastic modulus (Figure 3D; Table 2), representing mechanical behavior at a macro-scale. In this case, the substantial difference between the ash fraction (b=3.88) and the BV/TV exponent values (c=9.98) indicates a greater influence of the bone volume evolution over the total callus volume, encompassing both mineralized and non-mineralized phases, on the bone callus’ apparent mechanical properties during healing, compared to the influence of the ash fraction. This finding contrasts with previous reports on cortical and trabecular tissue in the literature, where ash fraction seems to have a significant greater influence on bone strength and modulus (Hernandez et al., 2001), possibly due to the less structural remodeling and time depending nature of healthy bone tissues.

There are several limitations associated with the methodology employed in this study. Firstly, it is expected that the mechanical, structural and chemical parameters analyzed *ex vivo* are not significantly influenced by differences in bone maturity of sheep during the regeneration period *in vivo* since the longest experiment (161 days) represents only around 4% of the animal life cycle. Secondly, the methods used for the indentation test (Oliver and Pharr, 1992) assume linear elastic behavior in the tissue, despite the possibility that the woven bone could exhibit viscoelastic behavior in some callus interzones, particularly during early stage of mineralization. However, the mechanical characterization performed in this study is focused on the mineralized phase, where the influence of the viscoelastic behavior should not be significant compared to a soft tissue (Blázquez-Carmona et al., 2021b). Using the Oliver-Pharr method in the evaluation of the nanoindentations also presents several limitations. The elastic modulus of surrounding tissue sub-domains, along with the presence of microcracks or defects, may influence the homogenized and averaged elastic modulus measured by the indenter (Kariem et al., 2015; Pastrama et al., 2018; Königsberger et al., 2022). Additionally, the use of different specimens for each time-point introduces an inevitable source of variation in the measurements. Despite this, the results appear to follow logical trends, and the power laws derived are significant. Finally, a higher number of animals would have enhanced the statistical significance of the conclusions of this study. As previously mentioned, the authors prioritized the use of an ovine animal model, whose results are crucial for construction of more realistic micromechanical models and whose conclusions can be readily extrapolated to human clinical cases.

In conclusion, this work contributes to the development of novel micromechanical models applicable to numerical simulations of bone regeneration processes. It also enhances the understanding of the relationship between chemical composition, structural morphology, and the mechanical properties of woven bone at different length scales by combining multiple *ex vivo* techniques: micro-CT, nanoindentation, Raman spectroscopy, and chemical and elemental analysis. Addressing the questions posed at the beginning of the discussion, woven bone exhibited a significant increase in bone volume, primarily controlled by the thickening and unification of trabeculae, alongside a simultaneous increase in apparent ash fraction, calcium, and phosphate content. The power law established for the bulk elastic modulus highlights a more significant influence of the evolving trabecular microarchitecture on the apparent mechanical behavior of the bone callus. At the tissue scale, the Raman mineral-to-matrix ratio increased over the analyzed regeneration period, reaching values and crystallinity levels comparable to those of cortical bone. However, the local elastic modulus did not increase sufficiently to reach cortical values, potentially due to the role of microporosities within the trabeculae. Power laws predicting the evolution of the woven bone elastic modulus at this scale demonstrated that Raman parameters correlate better with the elastic modulus than apparent mineral content measured through ash analysis and elemental studies. Therefore, we advocate for the combined use of Raman spectroscopy and nanoindentation in constructing future power laws for regenerating tissue, potentially strengthened by including a second parameter to account for structural features at the microscale.

## Data Availability

The original contributions presented in the study are included in the article/Supplementary Material, further inquiries can be directed to the corresponding author.
