# Supplementary material for: On the influence of structural and chemical properties on the elastic modulus of woven bone under healing
Source: Front Bioeng Biotechnol. 2024 Oct 1;12:1476473. doi: 10.3389/fbioe.2024.1476473 (PMC11473380; doi:10.3389/fbioe.2024.1476473)
Supplement: Supplementary file 1 [file DataSheet1.PDF]

Supplementary Material

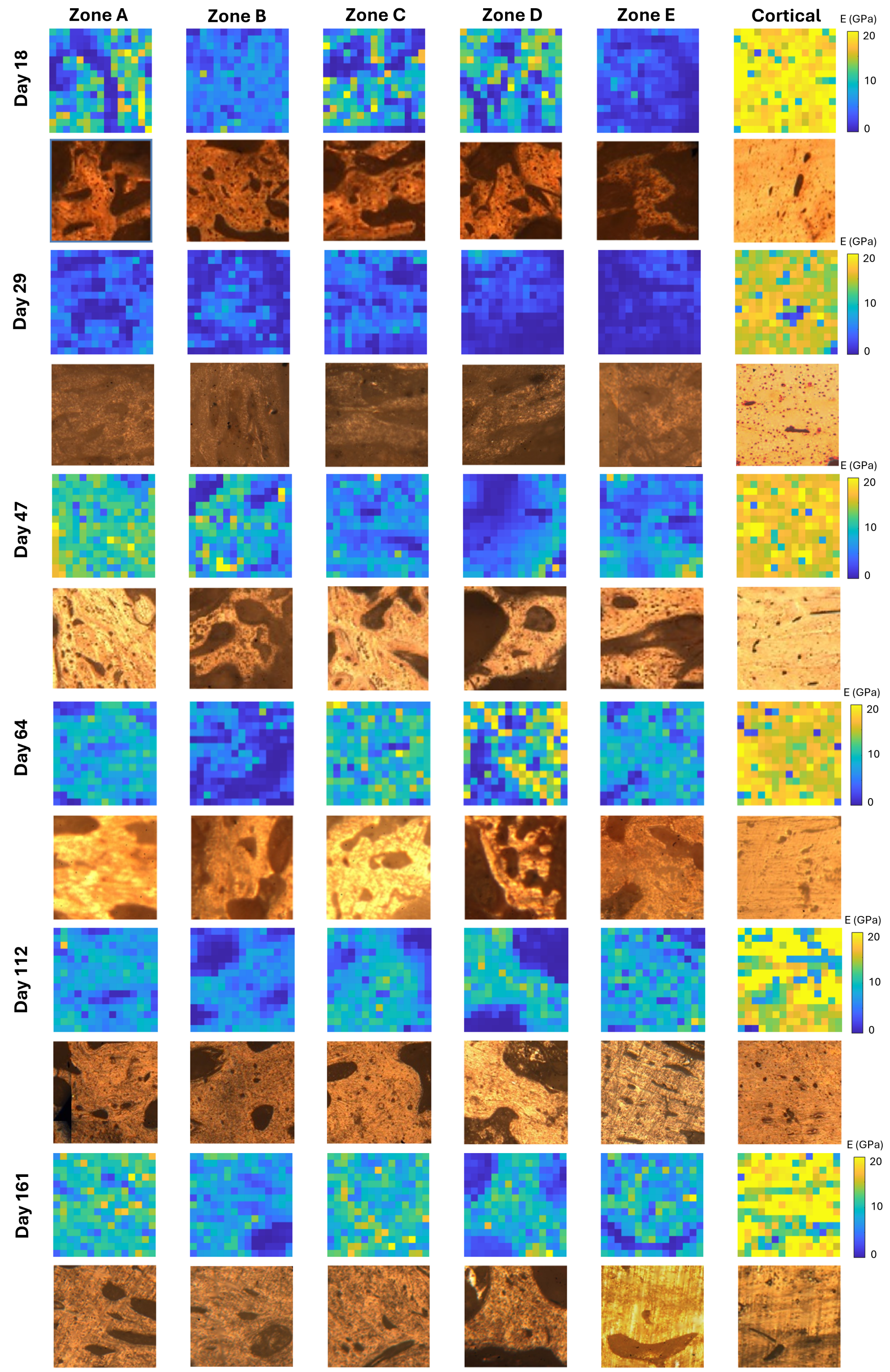

Figure S1. Nanoindentation maps of all analyzed zones of the bone callus samples.

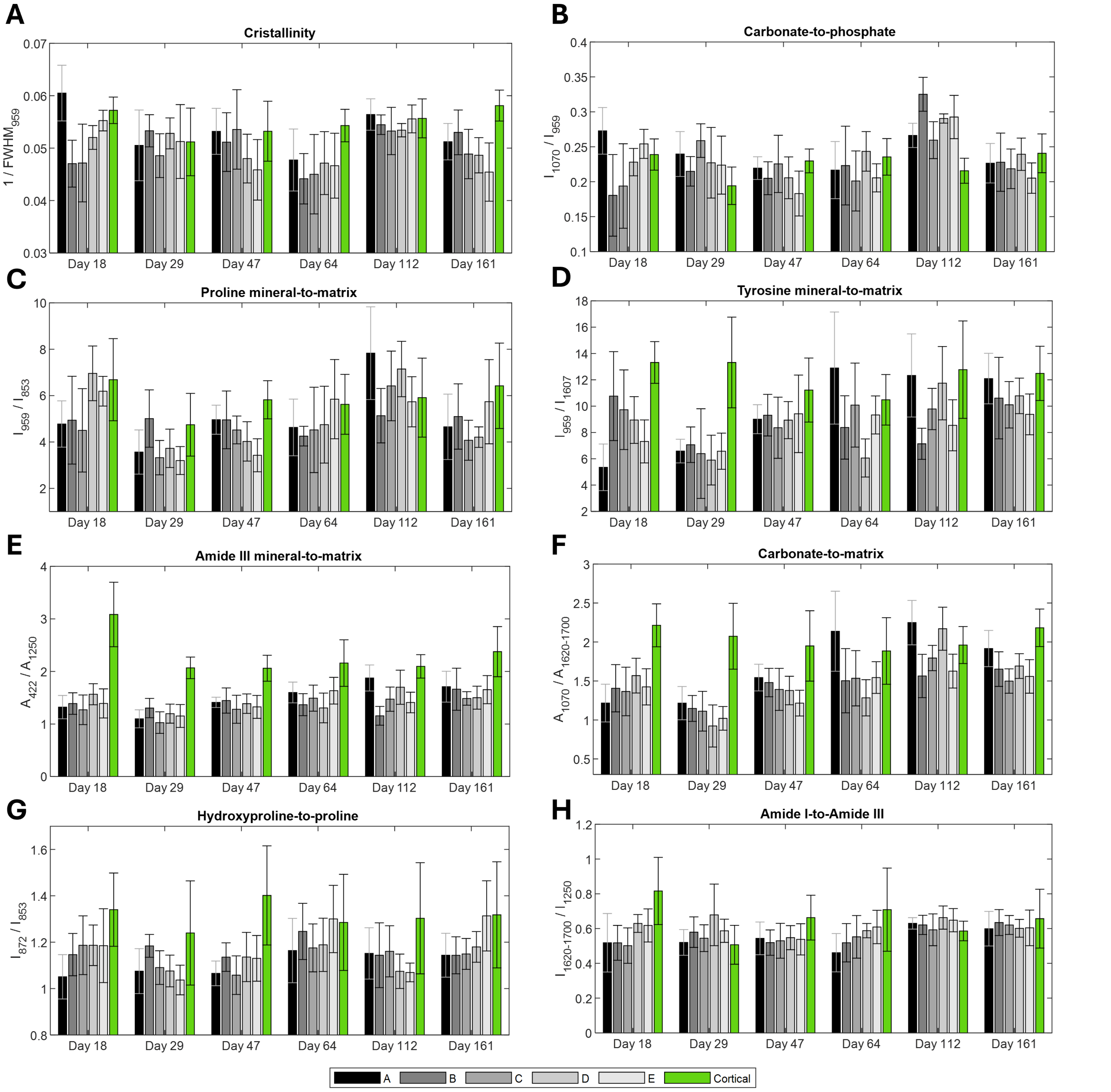

Figure S2. Evolution of the woven bone's chemical composition measured with Raman spectroscopy in each analyzed zone of the bone calluses: (A) crystallinity, (B) carbonate-to-phosphate, (C) proline mineral-to-matrix, (D) tyrosine mineral-to-matrix, (E) amide III mineral-to-matrix, (F) carbonate-to-matrix, (G) hydroxyproline-to-proline, and (H) amide I-to-amide II. Results of the cortical area of the callus (mean and standard deviation) is provided as a reference in green. I: maximum peak intensity, A: area under the peaks, FWHM: full width at half maximum. the subscript indicates the Raman shift position (cm^{-1}).

Table S1. Structural parameters measured in the woven bone samples by micro-CT: bone volume fraction (BV/TV), trabecular thickness (Tb.Th), trabecular separation (Tb.Sp), trabecular number (Tb.Nm), connectivity (Conn.D), degree of anisotropy (DA), and structural model index (SMI).

Sample	BV/TV	Tb.Th (mm)	Tb.Sp (mm)	Tb.Nm (mm^{-1})	Conn.D	DA	SMI
18 days	0.44	0.10	0.07	5.48	126.60	0.46	-4.53
29 days	0.47	0.12	0.15	3.55	42.06	0.49	-2.11
47 days	0.61	0.20	0.12	2.95	9.32	0.46	-2.38
64 days	0.62	0.28	0.17	2.20	7.98	0.50	-4.68
112 days	0.64	0.24	0.13	2.64	5.00	0.45	-4.54
161 days	0.68	0.29	0.13	2.33	2.46	0.49	-6.74

Table S2. Evolution of the woven bone's chemical composition measured with ash and elemental analysis: ash fraction, carbon percentage (C %), calcium percentage (Ca %), potassium percentage (K %), magnesium percentage (Mg %), sodium percentage (Na %), phosphorus percentage (P %), and strontium percentage (Sr %).

Sample	Ash fraction	C (%)	Ca (%)	K (%)	Mg (%)	Na (%)	P (%)	Sr (%)
18 days	28.90	0.16	45.91	0.48	0.57	1.80	23.45	0.02
29 days	15.47	0.23	35.71	0.71	0.51	2.70	20.03	0.02
47 days	46.01	0.15	47.24	0.34	0.89	1.59	24.16	0.02
64 days	40.80	0.37	43.51	0.53	0.57	2.43	22.49	0.03
112 days	53.31	0.36	46.76	0.15	0.73	1.08	23.12	0.01
161 days	56.72	0.65	47.95	0.13	0.44	1.09	23.39	0.01
